# Predicting Long-Term After-Effects of Theta-Burst Stimulation on Supplementary Motor Network Through One-Session Response

**DOI:** 10.3389/fnins.2020.00237

**Published:** 2020-03-27

**Authors:** Gong-Jun Ji, Jinmei Sun, Pingping Liu, Junjie Wei, Dandan Li, Xingqi Wu, Lei Zhang, Fengqiong Yu, Tongjian Bai, Chunyan Zhu, Yanghua Tian, Kai Wang

**Affiliations:** ^1^Department of Neurology, The First Affiliated Hospital of Anhui Medical University, Hefei, China; ^2^Department of Medical Psychology, Chaohu Clinical Medical College, Anhui Medical University, Hefei, China; ^3^Anhui Province Key Laboratory of Cognition and Neuropsychiatric Disorders, Hefei, China; ^4^Collaborative Innovation Centre of Neuropsychiatric Disorder and Mental Health, Hefei, China

**Keywords:** continuous theta-burst stimulation, functional connectivity, magnetic resonance imaging, transcranial magnetic stimulation, supplementary motor area

## Abstract

To understand the neural mechanism of repetitive transcranial magnetic stimulation (rTMS), the after-effects following one session or multiple days of stimulation have been widely investigated. However, the relation between the short-term effect (STE) and long-term effect (LTE) of rTMS is largely unknown. This study aims to explore whether the after-effects of 5-day rTMS on supplementary motor area (SMA) network could be predicted by one-session response. A primary cohort of 38 healthy participants underwent five daily sessions of real or sham continuous theta-burst stimulation (cTBS) on the left SMA. Resting-state functional magnetic resonance imaging (fMRI) data were acquired at the first (before and after the first stimulation) and sixth experimental day. The SMA connectivity changes after the first cTBS and after 5 days of stimulation were defined as STE and LTE, respectively. Compared to the baseline, significant STE and LTE were found in the bilateral paracentral gyrus (ParaCG) after real stimulation, suggesting shared neural correlates of short- and long-term stimulations. Region-of-interest analysis indicated that the resting-state functional connectivity between SMA and ParaCG increased after real stimulation, while no significant change was found after sham stimulation. Leave-one-out cross-validation indicated that the LTE in ParaCG could be predicted by the STE after real but not sham stimulations. In an independent cohort, the after-effects of rTMS on ParaCG and short- to long-term prediction were reproduced at the region-of-interest level. These imaging evidences indicate that one-session rTMS can aid to predict the regions responsive to long-term stimulation and the individualized response degree.

## Introduction

Repetitive transcranial magnetic stimulation (rTMS) is a powerful technique that could non-invasively modulate neural activity in human brain (Allen et al., 2007). It has been widely used to map brain function of healthy subjects or alleviate clinical symptoms for neuropsychological patients. However, high variability of rTMS after-effects has also been reported in both neuroscientific and clinical studies (Hamada et al., 2013; Yesavage et al., 2018). For instance, although continuous theta-burst stimulation (cTBS) was initially proposed as an inhibitory protocol (Huang et al., 2005), a study with a larger sample size (*n* = 52) indicated that only 42% subjects respond to the stimulation as expected (Hamada et al., 2013). But notably, the variability may change with the readout measures. Electroencephalography might represent a more thorough reflection of cortical excitability than motor evoked potentials (MEP) (Rocchi et al., 2018). Consistent with this variability in healthy subjects, another study observed that less than half of patients with major depression could achieve symptom remission after days of rTMS treatment (Yesavage et al., 2018). Here, we defined the after-effects induced by days of rTMS as long-term effect (LTE). Before rTMS could be recommended as a conventional therapy, more investigations are required to elucidate the neural mechanism and individualized after-effect prediction.

Based on the Faraday’s law of electromagnetic induction, TMS could induce an electrical field in the underlying brain tissues by generating a strong time-varying magnetic field. This electrical field drives currents in the cortical surface directly modulating electrical neuronal activation (Neggers et al., 2015). At the macro “neural systems” level, neuroimaging studies indicated that rTMS-induced effect can influence the activity of brain regions distant to the stimulation target (Valchev et al., 2015, 2016; Ji et al., 2017), suggesting a network mechanism (Bestmann and Feredoes, 2013; Sale et al., 2015; Hallett et al., 2017). By mapping whole-brain activity with high spatial resolution, functional magnetic resonance imaging (fMRI) provides a powerful approach to investigate rTMS effect in a network perspective (Sale et al., 2015; Bergmann et al., 2016). For brain disorders, the therapeutic mechanism of rTMS could be directly elucidated by comparing fMRI data before and after treatment. However, this paradigm requires a good combination of scientific and clinical resources. Alternatively, more studies turned to indirectly infer the treatment mechanism by assessing one-session rTMS effect on brain function. For instance, in a study on Parkinson’s disease (PD), meta-analysis on random control trials (RCTs) indicated that inhibitory rTMS on supplementary motor area (SMA) may improve the motor symptoms (Chou et al., 2015), but few fMRI studies investigated the functional changes after clinical treatment. On the contrary, numerous studies focused on the after-effects of one-session rTMS on motor network (Reithler et al., 2011; Di Lazzaro and Rothwell, 2014; Ji et al., 2017), which could be termed as short-term effect (STE). For instance, resting-state fMRI (RS-fMRI) study indicated that cTBS significantly decreased the functional connectivity of SMA target (Ji et al., 2017). Nevertheless, it is largely unknown whether this STE could be used to predict LTE, which is critical to bridge the findings of basic neuroscience and clinic treatment.

Although both STE and LTE indicated high inter-individual variability, few studies directly compared them. Using fMRI and MEP, Nettekoven et al. (2015) found that individuals who did not respond to one-session stimulation cannot be transformed into responders by increasing stimulation dose. In this study, we hypothesized that the responsiveness to rTMS is a reflection of the participant’s inherent and reliable traits (Hinder et al., 2014), and the LTE can be inferred from STE. To test this hypothesis, this study collected two resting-state fMRI datasets after one-session and 5 consecutive days of stimulations on the left SMA, respectively. We selected SMA as target because of its critical role in movement control. It was a potential effective rTMS target for alleviating symptoms of movement disorders (Shirota et al., 2013; Fox et al., 2014). We predicted that STE and LTE in SMA network have similar spatial distribution, and the LTE in functional connectivity could be individually predicted by their STE. Furthermore, independent data were collected to show the reproducibility of the relation between STE and LTE.

## Materials and Methods

### Subjects

A total of 54 participants without any neurological or psychiatric diseases were initially recruited in this study. Ultimately, 33 and 16 subjects completed the primary and secondary experiments, respectively. This study protocol was reviewed and approved by the Medical Ethics Committee of Anhui Medical University. All participants provided informed, written consent.

### Study Design

**FIGURE 1:**
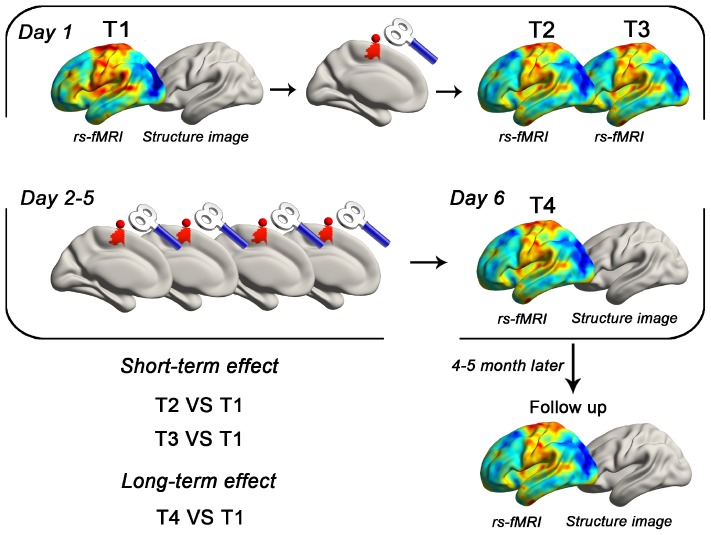
Schematic of the primary experiment. Using a between-subject design, each subject received real or sham cTBS for 5 consecutive days. The target area was defined as the superficial central point (MNI coordinates: −6, −6, 77; radius = 6 mm) of the left SMA proper in the Automated Anatomical Labeling template. RS-fMRI data were collected in five time points (T1, T2, T3, T4, and follow-up). T1 images were also obtained in the first and sixth day.

The primary experiment was designed as a single-blinded and between-subjects-based study (Figure 1). The participants were randomly assigned to real (*n* = 16) or sham (*n* = 17) groups, receiving cTBS for 5 days. At the first experiment day, one (T1) and two RS-fMRI (T2 and T3) sessions were performed before and after cTBS, respectively. STE would be estimated by comparing the data of T2/T3 to T1, while the LTE was estimated by comparing the RS-fMRI data at the sixth experiment day (T4) to T1. Structure images were acquired at the first and sixth experiment day as well. After 4 to 5 months, resting-state functional and structural images were collected to show the follow-up changes.

The second experiment was designed to reproduce the findings of the primary experiment. All participants (*n* = 16) received real cTBS for 5 consecutive days. Imaging data were acquired using the same parameters as the primary experiment.

### Neuronavigated Transcranial Magnetic Stimulation

Transcranial magnetic stimulation was performed using a MagStim Rapid^2^ stimulator (Magstim Company Ltd.) with a 70-mm air-cooled figure-of-eight coil. High-resolution anatomical images were acquired in the sagittal orientation using a three-dimensional brain-volume sequence (repetition/echo time, 8.16/3.18 ms; flip angle, 12°; field of view, 256 mm × 256 mm; 256 × 256 matrix; section thickness, 1 mm, without intersection gap; voxel size, 1 mm × 1 mm × 1 mm; 188 sections) for neuronavigation. The left SMA was defined as the target in the current study because of its potential in rTMS treatment for movement disorders (Le et al., 2013; Shirota et al., 2013; Chou et al., 2015; Eggers et al., 2015; Landeros-Weisenberger et al., 2015). A spherical image centered at the superficial central point (MNI coordinates: -6, -6, 77; radius = 6 mm) of the left SMA proper was transformed into each subject’s individual space by SPM12^1^ and TMStarget software (Ji et al., 2017). Then, the individualized target was imported into a frameless neuronavigation system (Brainsight; Rogue Research, Montreal, Canada). The coil was maintained horizontally pointing leftward, with the center of the coil positioned over the target (Zenon et al., 2015; Ji et al., 2017).

The cTBS protocol lasted 40 s and consisted of a burst of three pulses delivered at 50 Hz, which was repeated every 200 ms (at 5 Hz) for a total of 600 pulses. This 40-s protocol was repeated three times (1800 pulses in total) with two 15-min breaks (controlled by a stopwatch) (Nettekoven et al., 2014; Ji et al., 2017; Chen et al., 2018). cTBS was performed in a triple way to achieve accumulative after-effects (Nettekoven et al., 2014). Pulses were delivered at 70% of the resting motor threshold (RMT) (Nettekoven et al., 2014) that was defined as the lowest-intensity evoking MEP amplitudes of the first dorsal interosseus (>50 μV) in more than 5 of 10 consecutive trials. During RMT test, the coil was held approximately at a 45° angle away from the midline with the handle pointing backward and laterally. The electromyography signal was recorded using Ag/AgCl surface electrodes, amplified, digitized, and displayed by the Rogue EMG device.

Participants in the sham group received the same rTMS protocol and duration as the real rTMS group. The only difference was the usage of a sham coil (Magstim Company Ltd.) that produced a similar feeling on the participant’s scalp as the real coil but did not induce a current in the cortex. To assess the integrity of blinding, subjects were asked which intervention they had received at T4.

### RS-fMRI Data Acquisition

All MRI datasets were obtained at University of Science and Technology of China with a 3-T scanner (Discovery 750; GE Healthcare, Milwaukee, WI, United States). Foam padding and earplugs were applied to minimize head motion and scanner noise for all subjects. Participants were instructed to rest with their eyes closed without falling asleep during resting-state fMRI scanning. Functional images (217 volumes) were acquired using a single-shot gradient-recalled echo planar imaging sequence (repetition/echo time, 2400/30 ms; flip angle, 90°). Images of 46 transverse sections (field of view, 192 mm × 192 mm; 64 × 64 in-plane matrix; section thickness without intersection gap, 3 mm; voxel size, 3 mm × 3 mm × 3 mm) were acquired parallel to the anteroposterior commissure line. Each subject received MRI scanning for five times (T1, T2, T3, T4, and follow up). In the first experiment day, the stimulation and scanning (T1, T2, and T3) were performed in the morning. Immediately after stimulation, subjects were pushed into the MRI room for T2 and T3 by a compatible wheeled stretcher. The transfer time was recorded using a stopwatch.

### RS-fMRI Data Processing

Functional images were processed using the DPARSF^2^ (Chao-Gan and Yu-Feng, 2010), TMStarget^3^, REST^4^ (Song et al., 2011), and SPM12^5^. For preprocessing, we deleted the first five functional volumes, and then performed slice timing and realignment for the rest of the images. Structural images were then co-registered with these preprocessed functional images, and segmented into GM, WM, and cerebrospinal fluid (CSF) by Diffeomorphic Anatomical Registrations through Exponentiated Lie Algebra (DARTEL) (Ashburner, 2007). Normalized functional images were smoothed with a 4-mm full-width at half-maximum isotropic Gaussian kernel. Sources of spurious variance from each voxel’s time series were removed by (a) filtering temporal bandpass (0.01–0.1 Hz) and (b) regressing out nuisance signals including 24 head-motion parameters, and mean signals in the whole brain, white matter, and CSF. No subject had head motion exceeding 3 mm of translation or 3° of rotation during the fMRI acquisition.

## Statistical Analysis

### Group-Level rTMS After-Effects

The SMA network was defined by performing a seed-to-whole-brain functional connectivity analysis. The seed was the rTMS target in the left SMA (MNI coordinates −6, −6, 77; radius = 6 mm). Positive correlations were converted to *z* scores using the Fisher *r*-to-*z* transformation and tested by the one-sample *t* test. Both real and sham groups were included for producing between-group comparison mask. Specifically, we conducted one-sample *t* tests for each group (four conditions in total). Voxels that survived either test (uncorrected voxel level *P* < 0.05) were included as mask for between-group comparisons (paired *t* tests). This comparison was performed through a toolbox in SPM12, Statistic non-Parametric Mapping (SnPM) (Nichols and Holmes, 2002). To control the family-wise error (FWE) in multiple comparisons, we first set a voxel level threshold *P* < 0.01. Then, only clusters larger than a given volume would be reported as having survived the cluster-level correction, *P*_*corr*_ < 0.05.

### Individualized LTE Prediction

The predicting value of STE for LTE was estimated by leave-one-out cross-validation. Briefly, we sequentially selected one subject as a test, and the others as training data. In the training data, resting-state functional connectivity (RSFC) changes of the target (i.e., STE) were estimated by comparing the post- and pre-rTMS conditions. Voxels with significant STE (*P*_*corr*_ < 0.05) were defined as ROIs. RSFC alterations in these ROIs were correlated between short-term (T2/T3 minus T1) and long-term (T4 minus T1) conditions. Based on the information of the voxel with the highest correlation coefficient, a linear function between STE and LTE could be established. Then, predicted LTE of the test subject could be computed through the function and STE. Finally, Pearson’s correlation was performed between the real and predicted LTE across subjects.

## Results

### Characteristics of Participants

Five measures (age, gender, education, RMT, and interval) at baseline were compared within the primary cohort (real vs. sham), and no significant difference was found (Table 1). The interval refers to the period from the end of cTBS to the beginning of fMRI scanning at the first experiment day. These measures were also compared between the real groups of the primary and secondary cohorts. No significant difference was found either (Table 1).

**TABLE 1:** Characteristics of participants in the primary and second cohorts.

	**Primary cohort**			**Second cohort**	
		
	**Real (*n* = 16)**	**Sham (*n* = 17)**	**Statistics/*P*^a^**	**Real (*n* = 16)**	**Statistics/*P*^b^**
Age (years)	21.6 ± 0.48	20.9 ± 0.71	0.78/0.44^c^	20.4 ± 0.51	1.68/0.10^c^
Gender (male/female)	6/10	11/6	0.17^d^	11/5	0.16^d^
Education (years)	15.4 ± 0.52	14.5 ± 0.50	1.25/0.22^c^	14.7 ± 0.44	1.09/0.28^c^
RMT (%)	58.4 ± 1.58	59.4 ± 1.26	0.49/0.63	58.4 ± 1.94	0.0/>0.99^c^
Interval ^e^	192.9 ± 5.42	194.9 ± 7.16	0.22/0.83^c^	193.2 ± 2.72	0.052/0.96^c^
Follow-up (days)	140.1 ± 4.61	138.8 ± 4.33	0.20/0.85^c^	133.4 ± 2.87	1.23/0.23^c^

*Data are represented as mean ± SEM. ^*a*^The analysis was performed between the two groups of the primary cohort. ^*b*^The analysis was performed between the real groups of the primary and secondary cohorts. ^*c*^Two-sample *t* test. ^*d*^Fisher’s exact test. ^*e*^Interval depicts the period from the end of cTBS to the beginning of functional MRI scanning at the first experiment day.*

In the primary cohort, around half of the participants in the real (50%, 8 in 16) and sham (41%, 7 in 17) group correctly guessed which group they belong to (*Fisher’s* exact test, *P* = 0.73). This ratio in the second cohort is 41% (7 in 17), similar to the real group in the primary cohort (*Fisher’s* exact test, *P* = 0.72).

### Group-Level After-Effects in the Primary Cohort

**FIGURE 2:**
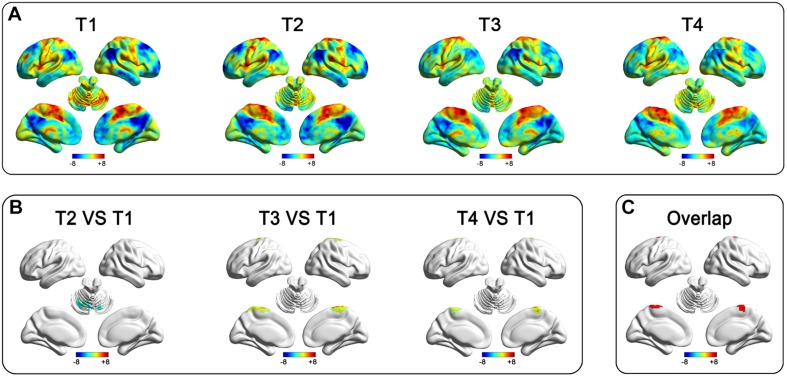
After-effect of rTMS on functional connectivity in the real group of the primary cohort. Functional connectivity patterns of the target (left SMA) before and after stimulation **(A)**. Functional connectivity decreased in the bilateral cerebellum at T2 and increased in the bilateral paracentral gyrus at T3 and T4 after stimulation **(B)**. The rTMS after-effect at T3 and T4 have a spatial overlap at the bilateral ParaCG **(C)**.

To show the SLE and LTE, we firstly analyzed the after-effects of rTMS at T2, T3, and T4, respectively. Compared to the pre-rTMS state (T1), the real group showed decreased RSFC in the bilateral cerebellum at T2 (Table 2 and Figures 2A,B), and increased RSFC in the bilateral paracentral gyrus (ParaCG) at T3 and T4 (Table 2 and Figures 2A,B). The Dice value for clusters at T3 and T4 was 0.4 (Figure 2C). Notably, the peak voxel in T4 was significant at T3 (*t* = 4.03, *P* = 0.001), and vice versa (*t* = 2.11, *P* = 0.05).

**TABLE 2:** Functional connectivity alterations after real cTBS in the primary cohort.

**Contrast**	**Brain regions**	**MNI coordinates**	**Brodmann area**	**Voxel number**	**Peak *t* value**
T2 vs. T1	Cere B.	15 -75 -18	–	128	5.55
T3 vs. T1	ParaCG B.	-15 -15 72	4	410	6.36
T4 vs. T1	ParaCG B.	9 -21 72	4	126	4.91

*Cere B., bilateral cerebellum; MNI, Montreal Neurological Institute; ParaCG B., bilateral paracentral gyrus.*

The aim of this study was to predict LTE. To this end, we should first demonstrate that the findings in T4 were not induced by placebo effect. Although no significant functional alteration was found in sham group at T4, a direct real-sham comparison was still necessary. This was performed in both voxel and ROI level.

The voxel-wise ANOVA showed significant interaction effect (group [real and sham] by time [T1 and T4]) within significant regions at T4 in the real group. A significant RSFC increase was found in the real group, but not the sham group (see Supplementary Material).

**FIGURE 3:**
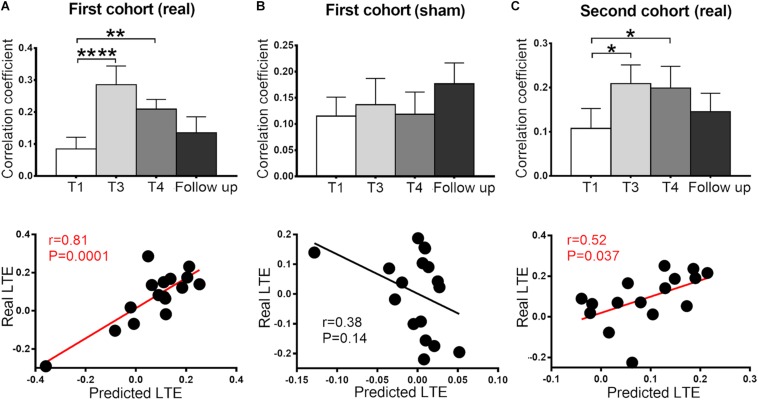
Functional connectivity between SMA and ParaCG at four time points (T1, T3, T4, and follow-up) and cross-time prediction in the two cohorts. In the primary cohort, the connectivity significantly increased at T3 and T4 in the real **(A)** but not sham **(B)** group. This alteration was reproduced in the second cohort **(C)**. No significant difference was found between the baseline and follow-up in all three groups. The predicted LTE was positively correlated with the real values in real groups **(A,C)**, but not sham group **(B)**. Error bars indicate SEM. *P < 0.05, **P < 0.01, and ****P < 0.0001. None of the values were outliers that exceeded three standard deviations of the mean.

For ROI-level analysis, a sphere ROI at the ParaCG (centered at the peak voxel at T4, radius = 3 mm) was used for the following sham-control analyses. We compared the RSFC of ParaCG ROI between real and sham groups at four time points (T1, T3, T4, and follow-up) using two-way ANOVA. Main effect was significant for time (*F* = 4.51, *P* < 0.01) but not group (*F* = 0.72, *P* = 0.40). Interaction effect between group and time was significant (*F* = 4.67, *P* = 0.004). Compared to the baseline at T1, a higher RSFC between the ParaCG ROI and SMA was found at T3 (*t* = 4.61, *P* < 0.0001), T4 (T1, *t* = 2.85, *P* = 0.005), but not follow-up (*t* = 1.17, *P* = 0.25) in the real group (Figure 3A). No significant changes were found in the sham group (T3 vs. T1, *t* = 0.51, *P* = 0.61; T4 vs. T1, *t* = 0.09, *P* = 0.93; follow up vs. T1, *t* = 1.46, *P* = 0.15; Figure 3B).

### Individual After-Effect Prediction in the Primary Cohort

The after-effect at T3 and T4 spatially overlapped at the bilateral ParaCG (Figure 2C), suggesting that STE and LTE have similar regions in responding. To predict the individualized RSFC changes at the T4, leave-one-out cross-validation was used. At each time of leave-one-out, a significant cluster at ParaCG was identified (T3 vs. T1, cluster level *P*_*corr*_ < 0.05). The linear function between STE and LTE in this cluster was used to predict LTE of the test subject. Correlation analysis indicated that the predicted LTE values were positively correlated with the real values (*r* = 0.81, *P* = 0.0001; Figure 3A).

Since voxel-level analysis for the sham group did not show significant RSFC change at T3 or T4 (cluster level *P*_*corr*_ < 0.05), the leave-one-out prediction was performed with predefined ParaCG ROI from the real group. The predicted and real LTE did not show significant correlation (*r* = 0.38, *P* = 0.14; Figure 3B).

### Independent Validation

For the analysis of the primary cohort, we compared the SMA-to-whole brain RSFC between post- and pre-rTMS conditions. Neither T3 nor T4 showed significant alteration at the voxel level (cluster level *P*_*corr*_ < 0.05). However, analysis on the ParaCG ROI (from the primary cohort) indicated significant findings. As compared to T1, RSFC of the ParaCG was significantly higher in T3 (*t* = 2.64, *P* = 0.02) and T4 (*t* = 2.93, *P* = 0.01), but not in the follow up (*t* = 1.02, *P* = 0.32). By leave-one-out approach, we computed the predicted RSFC value of ParaCG in each subject. Correlation analysis indicated a positive correlation between predicted and real LTE in this second cohort (*r* = 0.52, *P* = 0.037) (Figure 3C).

## Discussion

To investigate the association between the after-effects of short- and long-term rTMS, this study collected resting-state fMRI data for participants around one-session and 5 days of rTMS. In the primary cohort, we found that both short- and long-term stimulations increased the RSFC between the SMA target and bilateral ParaCG. Leave-one-out cross-validation indicated that the LTE predicted by STE was significantly correlated with the real values. These findings were not significant in the sham group, but reproduced in the validation cohort receiving real rTMS. In summary, this study suggests that the region responsive to long-term rTMS and the individualized response degree could be predicted by that of one-session stimulation.

### Group-Level After-Effects

Functional MRI studies for TMS could be roughly categorized into three classes that focused on immediate (Chen et al., 2013), short-term (Ji et al., 2017), and long-term (Chen et al., 2018) after-effects, respectively. Interleaved TMS and fMRI acquisition could reveal the immediate after-effect seconds after stimulations. By this approach, Vink et al. (2018) found that stimulations of the dorsal prefrontal cortex could trigger the activity of subgenual anterior cingulate cortex, which is a critical region in rTMS treatment for depression (Fox et al., 2012). It suggests that immediate effect of TMS may explain the mechanism of long-term treatment.

Here, we directly associated the short- and long-term after-effects of rTMS on SMA network. Using the same cTBS sequence, a previous study identified decreased RSFC in the inferior frontal gyrus and SMA regions (Ji et al., 2017), while the current study found increased RSFC in the ParaCG. Two factors may explain this discrepancy in STE. Firstly, the current study overcame previous limitations. All participants were transferred into the MRI room by an MRI-compatible wheeled stretcher to avoid movement-induced interference. Secondly, the interval between rTMS and fMRI scanning is 5.1 min in a previous study (Ji et al., 2017), while that in the current study, it is 3.2 and 11.9 min for T2 and T3, respectively. Different response patterns among these three intervals implicate that nodes in the target network may have distinct response time to rTMS.

Similar to the STE, increased RSFC was found at the ParaCG after 5 days of active stimulation. This cross-time similarity in response region is consistent to the findings on immediate and long-term after-effects (Vink et al., 2018) and may be helpful in developing clinical therapies. The gold standard for novel treatment usually requires long-term clinical trial with large samples. This is a resource-intensive approach for demonstrating novel rTMS protocols. Although this step cannot be omitted before clinical application, our findings suggest that a simple test for the STE may screen out protocols with effective LTE. More specifically, the STE of novel rTMS protocols may predict whether their long-term application could restore the RSFC biomarker and ultimately alter clinical symptoms. Our follow-up findings indicated that the LTE in RSFC changes was transient and decreased to baseline a few months later. Further studies are required to show the shortest washout period of long-term rTMS.

### Individual Prediction

Although rTMS protocols may stably increase or decrease the neural excitability at the group level, high variability of the after-effect was also reported across subjects (Hamada et al., 2013; Yesavage et al., 2018). In these studies, MEP is frequently used as a readout measure. However, MEP was also affected by factors except cortical excitability, such as spinal motoneuron excitability. In contrast, measures originated from cortices may be a better readout than MEP, such as EEG and fMRI (Rocchi et al., 2018). In this study, we associated the STE and LTE using functional connectivity. By leave-one-out cross-validation, we found a significantly positive correlation between STE and LTE in the real group, but not the sham group. Given the poor reproducibility of most neuroimaging findings (Eklund et al., 2016), we additionally performed the prediction analysis in an independent cohort. The correlation between predicted and real LTE was well reproduced. This positive prediction is consistent to previous findings that the responsiveness of subjects to rTMS is similar between one and three sessions of TBS (Nettekoven et al., 2015). This within-subject consistency may be explained by the stability of the interneuron networks that were recruited during stimulation (Hamada et al., 2013). Thus, these neuroimaging findings may be generalized to clinical prediction. The symptom improvement after long-term rTMS therapy may be inferred at the beginning of treatment, such as the response to the first stimulation session.

### Limitations

Several limitations of the current study should be noted. Firstly, this is a neuroimaging study without behavior estimation. It would be interesting to investigate which kind of motor ability would be modulated with the RSFC alteration. Secondly, the rTMS after-effect in the primary cohort was validated in the second cohort by ROI-based analysis but not voxel-wise comparison. This is probably related to the small sample size and high after-effect variability between subjects (Nettekoven et al., 2015). Larger sample size is necessary to clarify the baseline characteristics and short-term response variability (Hamada et al., 2013). Thirdly, the figure-of-eight coil can successfully stimulate the superior part of SMA, while the medial part of SMA was largely unaffected. For future study, it would be interesting to test whether stimulating the medial part using a double cone coil can induce different after-effects (Georgiev et al., 2016; Mendez et al., 2017). Fourthly, the duration of our long-term stimulation is shorter than clinical rTMS treatment. For instance, guideline for depression treatment is 6 to 8 weeks (Blumberger et al., 2018). The number of response regions may be increased with the dose of stimulations. As a result, the STE may only predict part of the response region after long-term treatment. Because of the potential risk of applying longer stimulation for healthy subjects, this issue can only be addressed in patients that need rTMS treatment. Finally, the voxel with the highest LTE at T4 was close to the cortical target. The Euclidean distances between target and the two ParaCG peak points at T3/T4 were 13 and 22 mm, respectively. Although single stimulation with high strength (>RMT) only induced activation within 1-mm distance (Romero et al., 2019), the spatial extent of cTBS is still undetermined. Addressing this issue is important to explain to what extent the LTE in ParaCG was induced directly by cTBS.

## Conclusion

This study associated the after-effects of short- and long-term rTMS on SMA network. At the group level, both one-session and 5-day stimulations exclusively increased the RSFC between SMA and ParaCG. At the individual level, the 5-day after-effects could be predicted by an individual’s alteration after one-session stimulation. These imaging evidences indicated that one-session rTMS findings could predict the region’s response to long-term stimulations, as well as the individualized response degree. It suggests shared neural mechanisms between short- and long-term rTMS. Future rTMS studies on patients may further investigate whether the STE in neuroimaging could be a predictor for screening rTMS-sensitive subjects before the end of long-term treatment.

## Data Availability Statement

The datasets generated for this study are available on request to the corresponding author.

## Ethics Statement

The studies involving human participants were reviewed and approved by the Anhui Medical University. The patients/participants provided their written informed consent to participate in this study.

## Author Contributions

G-JJ and KW: full access to all the data in the study and take responsibility for the integrity of the data and the accuracy of the data analysis. G-JJ, JS, LZ, DL, and JW: study concept and design, acquisition, analysis, or interpretation of data. G-JJ, FY, CZ, XW, and TB: administrative, technical, or material support. CZ, YT, and KW: study supervision and obtained funding. All authors listed have made substantial, direct, and intellectual contribution to the work, and approved it for publication.

## Conflict of Interest

The authors declare that the research was conducted in the absence of any commercial or financial relationships that could be construed as a potential conflict of interest.

## Footnotes

1 www.fil.ion.ucl.ac.uk/spm

2 http://rfmri.org/DPARSF

3 http://www.brainhealthy.net

4 www.restfmri.net

5 http://www.fil.ion.ucl.ac.uk/spm
