# Supplementary material for: Predicting Long-Term After-Effects of Theta-Burst Stimulation on Supplementary Motor Network Through One-Session Response
Source: Front Neurosci. 2020 Mar 27;14:237. doi: 10.3389/fnins.2020.00237 (PMC7124138; doi:10.3389/fnins.2020.00237)
Supplement: Supplementary file 1 [file Data_Sheet_1.doc]

**Supplementary Material for**

# Predicting long-term after-effects of theta-burst stimulation on supplementary motor network through one-session response

Gong-Jun JI, Jinmei Sun, Junjie Wei, Dandan Li, Xingqi Wu, Lei Zhang, Fengqiong Yu, Chunyan Zhu, Yanghua Tian, Kai Wang*

*Corresponding author. E-mail: [wangkai1964@126.com](mailto:wangkai1964@126.com)


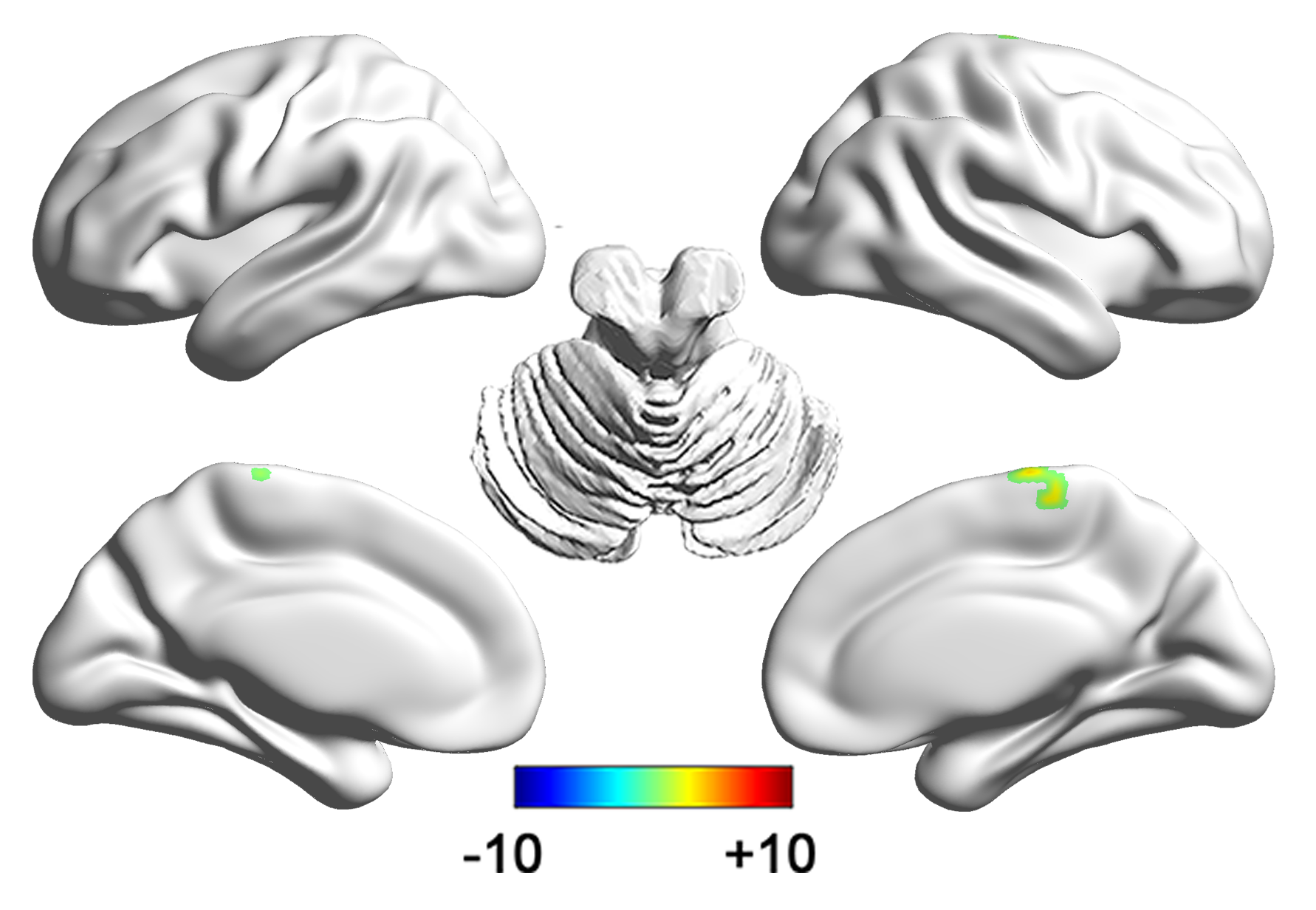


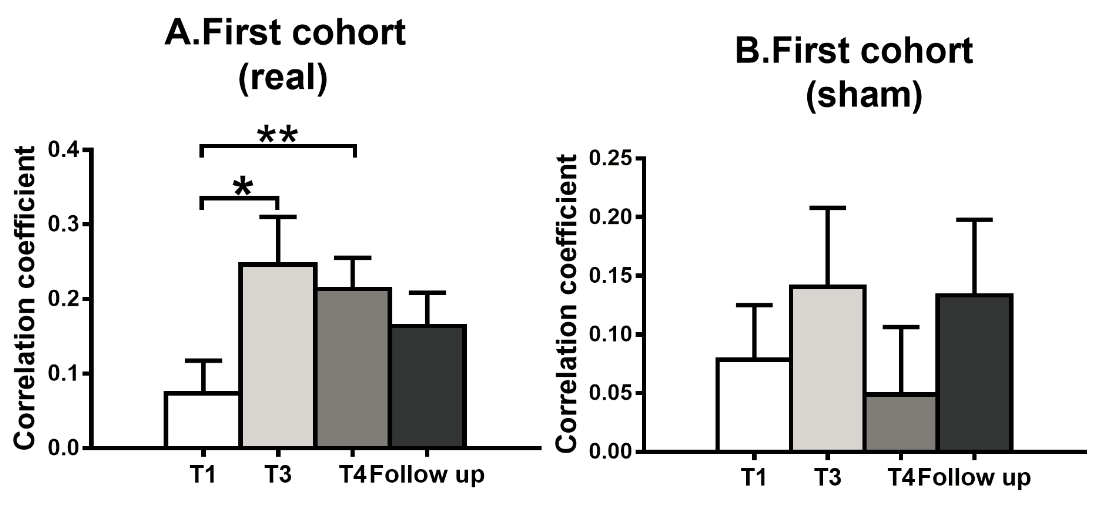
Fig S1. Significant interaction effect on functional connectivity between real and sham groups at two points (T1 and T4). Functional connectivity increased in the right paracentral gyrus.

Fig S2. Signal of the peak voxel from the Fig. S1 was extracted and compared between conditions by paired t test. T3 (t=2.75, P=0.02) and T4 (t=3.85, P=0.002) in real group indicated significant rTMS effect. No significant change was found in the sham group.
